# Effectiveness of cetuximab as preemptive postsurgical therapy for oral squamous cell carcinoma patients with major risk: a single‐center retrospective cohort study

**DOI:** 10.1007/s10637-021-01062-0

**Published:** 2021-01-15

**Authors:** Chonji Fukumoto, Yuta Sawatani, Ryo Shiraishi, Manabu Zama, Michiko Shimura, Tomonori Hasegawa, Yuske Komiyama, Atsushi Fujita, Takahiro Wakui, Hitoshi Kawamata

**Affiliations:** 1 Department of Oral and Maxillofacial Surgery, Dokkyo Medical University School of Medicine, 880 Kitakobayashi, Mibu, Tochigi, Shimo-Tsuga 321-0293 Japan; 2 Department of Dentistry, Oral and Maxillofacial Surgery, Sano Kosei General Hospital, 1728 Horigome, Sano, Tochigi 327-8511 Japan

**Keywords:** Oral squamous cell carcinoma, Preemptive postsurgical therapy, Cetuximab, Postsurgical treatment, Major risk

## Abstract

A retrospective cohort study was performed to investigate the effectiveness of preemptive postsurgical therapy with cetuximab for patients with a major risk of recurrence or metastasis after clinical complete resection of primary oral squamous cell carcinoma (OSCC). The study period was from 2007 to 2019 for patients treated at the Department of Oral and Maxillofacial Surgery, Dokkyo Medical University School of Medicine. OSCC patients with major risk (n = 88) in the follow-up period were divided into groups with no postsurgical treatment (NP group), with standard postsurgical treatment (SP group), and with postsurgical treatment including cetuximab (CP group), and prognosis were compared among those groups. The 5-year overall survival rate was significantly higher in patients who received postsurgical treatment with cetuximab (CP) compared to that in the other two groups ((CP vs. NP, p = 0.028; CP vs. SP, p = 0.042). Furthermore, we performed multivariate analysis to evaluate the effects of the main components of the treatment. Among CDDP, radiotherapy, and cetuximab, only cetuximab significantly contributed to improved survival by univariate analysis (crude HR:0.228, 95%CI:0.05–0.968, p = 0.045). cetuximab also showed the same tendency in multivariate analysis, although p value did not reach significant level (Adjusted HR: 0.233, 95%CI: 0.053–1.028, p = 0.054). The results suggest that the postsurgical treatment with cetuximab as a preemptive postsurgical therapy after complete surgical resection of a visible tumor is considerably effective for OSCC patients with major risk, in other words, invisible dormant metastasis.

## Introduction

Oral squamous cell carcinoma (OSCC) is mainly treated with surgery in combination with chemotherapy (including molecular-targeted drugs), immune checkpoint inhibitors, and radiotherapy. These treatments have improved overall survival (OS) in patients with OSCC, but local recurrence, cervical lymph node metastasis, and distal metastasis may still occur after initial clinical complete resection.

The guidelines for head and neck cancer of the National Comprehensive Cancer Network (NCCN) include extranodal extension, positive margins, close margins, pT3 or pT4 primary, pN2 or pN3 nodal disease, nodal disease in levels IV or V, perineural invasion, vascular invasion, and lymphatic invasion as adverse features (AFs) for a poor postsurgical outcome [1]. Among these AFs, we included extranodal extension, positive margins, close margins, pN2 or pN3 nodal disease, and nodal disease in levels IV or V, in addition to Yamamoto-Kohama (Y-K) mode of invasion [2, 3] Y-K4C and Y-K4D, in our definition of major risk factors for recurrence or metastasis. The Y-K mode of invasion has five stages (1, 2, 3, 4C, 4D) for the pathological grade of malignancy [2, 3] based on the shapes of tumor-cell cords at the tumor-host interface. A correlation between stage and outcome has been shown [2–4]. Of the other AFs, perineural invasion was rarely identified in OSCC. Then we recognized that clinical meaning of perineural invasion in OSCC was low. Vascular invasion and lymphatic invasion were also excluded from our definition of major risk factors because our previous basic and clinical research on metastasis suggested that histopathologically detected vascular invasion and lymphatic had little meaning for metastasis formation [5–9]. We started to use our definition of major risk factors for recurrence or metastasis from 2014.

The NCCN guidelines suggest chemotherapy after surgery for OSCC patients with AFs, with platinum-based chemotherapy and/or radiation used as the first line treatment [1]. However, due to the general condition of patients with advanced OSCC, use of standard platinum-based chemotherapy is often difficult, since most of such patients show malnutrition and hypofunction of the lung, liver, and kidney [10]. For this reason, the guidelines suggest that the treatment should be personalized.

cetuximab is an antibody to epidermal growth factor receptor (EGFR) that is used for treatment of metastatic colorectal cancer, metastatic non-small cell lung cancer, and head and neck cancer. We have suggested that the main effect of cetuximab in patients with OSCC may be immunological, such as antibody-dependent cellular cytotoxicity (ADCC), rather than signaling blockade [11]. In OSCC patients with local recurrence, lymph node metastasis, or distal metastasis, we hypothesized that dormant cancer cells that could not be detected before or during initial treatment might grow after the primary treatment and develop into a visible tumor. Therefore, from 2014, we have added treatment with cetuximab within 3 to 6 months after complete surgical resection of a visible tumor in order to attack the dormant invasive or metastasized cancer cells as preemptive postsurgical therapy for the OSCC patients with major risk, in other words, OSCC patients with invisible metastasis. Here, we report a retrospective cohort study of the effectiveness of the preemptive postsurgical therapy with cetuximab for OSCC patients with major risk after complete surgical resection of a visible tumor.

## Patients and methods

### Data sources

A retrospective cohort study was performed for patients with OSCC treated from 2007 to 2019 at the Department of Oral and Maxillofacial Surgery, Dokkyo Medical University School of Medicine. Data were obtained from electronic medical records. The study design was approved by the Medical Ethical Research Committee of Dokkyo Medical University Hospital (approval ID R-22-12J).

### Definition of the patients with major risk

The AFs of extranodal extension, positive margins, close margins, and pN2 or pN3 nodal disease in the NCCN Guidelines for Head and Neck Cancers [1], and Y-K4C or Y-K4D stage [2–4] were defined as major risk factors. If a patient fulfilled at least one of these factors, the patient was included as the patient with major risk.

### Patients

Patients with major risk (n = 88) were identified among patients who underwent surgery for primary OSCC during the study period. In patients with a resected tumor close to the surgical margin (within 5 mm) or with a tumor on the surgical margin, additional resection was performed within 1 week after recognition of the status of surgical margin. Therefore, complete resection of the visible tumor with safety margin was confirmed in all patients, although we enrolled these patients as the patients with major risk. The 88 patients were divided into groups with no postsurgical treatment (NP group), with the standard postsurgical treatment in the NCCN guidelines (SP group), and with postsurgical treatment including cetuximab (CP group). The 5-year OS and disease-free (DF) rates were evaluated in each group as outcomes. Multivariate analysis of outcomes and events was performed with cetuximab, cisplatin (CDDP), and radiotherapy as potential confounding factors. The cancer staging was performed by the UICC TNM Classification of Malignant Tumours, 8th ed [12].

### Statistical analysis

Descriptive analyses were performed for the baseline demographics and clinical factors in the 88 OSCC patients with major risk. A chi-squared test or Fisher exact test was used to compare categorical variables between the groups. The 5-year OS and DF rates were analyzed in each group using Kaplan-Meier survival analysis. To obtain hazard ratios (HRs) for mortality and related factors (cetuximab, CDDP, radiation) in OSCC, univariate and multivariate analyses were performed with a Cox proportional hazard model. Two-tailed*P* values of < 0.05 were considered to be significant. IBM SPSS ver. 24.0 (IBM SPSS, Inc., Tokyo, Japan) was used for all statistical analyses.

## Results

### Characteristics and treatment of OSCC patients with major risk

The characteristics and treatment of the 88 OSCC patients are shown in Table 1. The patients included 60 males (68.2%) and the median age was 65 years. Primary site, T stage, N stage, and cancer stage in all patients are shown. The most frequent primary site was tongue (47 patients, 53.4%), followed by lower gingiva (18 patients, 20.5%). In pathologically, patients with pT4a (47 patients, 53.4%) at the pT stage, pN2b (20 patients, 22.7%) and pN3b (25 patients, 28.4%) at the pN stage, and pStage 4a (37 patients, 42.0%) and 4b (25 patients, 28.4%) were frequently observed, because we selected the patients with major risk in this study. Extranodal expansion (3 patients, 3.4%), positive or close margins (10 patients, 11.4%), pN2 or pN3 (13 patients, 14.8%), YK-4C or YK-4D (37 patients, 42.0%) was picked up as major risk factors.

**Table 1:** Characteristics of patients with OSCC with major risk factors (n = 88)

Item	Value	
Sex, male, n (%)	60	(68.2)
Age, mean (SD) y	63.7	(13.6)
Age, median y	65	
Age group, n (%)		
< 65 y	40	(45.5)
≥ 65 to < 75 y	30	(34.1)
≥ 75 y	18	(20.5)
Primary site, n(%)		
Tongue	47	(53.4)
Lower gingiva	18	(20.5)
Upper gingiva	8	(9.1)
Buccal mucosa	8	(9.1)
Oral floor	4	(4.5)
Lip	1	(1.1)
Palate	2	(2.3)
Pathological T stage, n(%)		
T1	3	(3.4)
T2	19	(21.6)
T3	19	(21.6)
T4a	47	(53.4)
Pathological N stage, n(%)		
N0 (includes cases with local resection only)	32	(36.4)
N1	9	(10.2)
N2b	20	(22.7)
N2c	2	(2.3)
N3b	25	(28.4)
Pathological Stage, n(%)		
Stage 1	3	(3.4)
Stage 2	10	(11.4)
Stage 3	13	(14.8)
Stage 4a	37	(42.0)
Stage 4b	25	(28.4)
Major risk factor, n (%) overlapping distribution		
Extranodal extension	3	(3.4)
Positive or close margins	10	(11.4)
pN2 or pN3	13	(14.8)
YK-4C or YK-4D	37	(42.0)
Treatment, n (%)		
Surgery only (NP group)	29	(33.0)
Surgery + standard postsurgical treatment (SP group)	38	(43.2)
Chemotherapy with Cisplatin	8	
Radiation	11	
Chemoradiation with Cisplatin	19	
Surgery + Cetuximab-combined postsurgical treatment (CP group)	21	(23.9)
Chemotherapy with Cetuximab + Paclitaxel	12	
Chemoradiation with Cetuximab	6	
Chemoradiation with Cetuximab, and chemotherapy with Cetuximab + Paclitaxel	3	

NP: No Postsurgical treatment

SP: Standard Postsurgical treatment

CP: Cetuximab-combined Postsurgical treatment

The NP group (no postsurgical treatment) included 29 patients (33.0%). The SP group (standard postsurgical treatment based on the NCCN guidelines) included 38 patients (43.2%) who received radiotherapy alone, cisplatin alone, or concurrent chemoradiotherapy with cisplatin. The CP group (postsurgical treatment including cetuximab) included 21 patients (23.9%) who received cetuximab + Paclitaxel (PTX), combination bioradiotherapy (BRT) of radiotherapy and cetuximab, or cetuximab /PTX therapy after BRT.

### Characteristics and prognoses in the patients with different postsurgical treatment

**Fig. 1:**
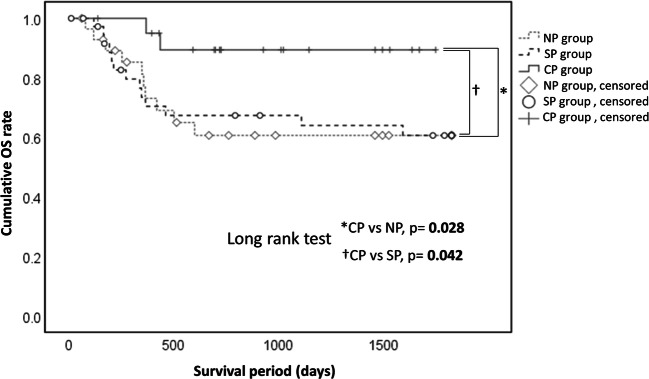
Cumulative overall survival (OS) rate in patients with OSCC. The 5-year OS rate in the CP group (90.5%) was significantly higher than those in the NP (62.1%, P = 0.028) and SP (65.8%, P = 0.042) groups

**Fig. 2:**
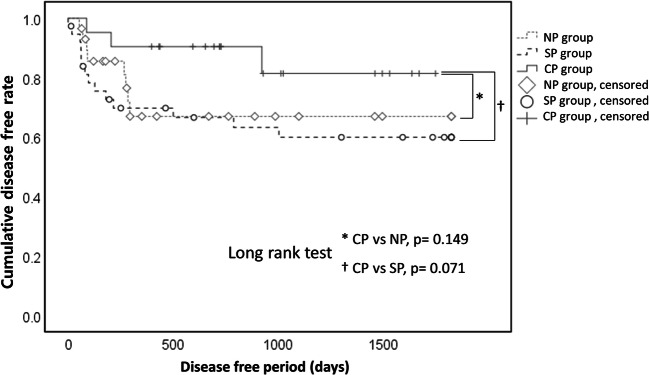
Cumulative disease free (DF) rate in patients with oral SCC. The 5-year DF rate in the CP group (85.7%) was higher than those in the NP (72.4%, P = 0.149) and SP (63.2%, P = 0.071) groups, but the differences were not significant

The characteristics and prognoses in the NP, SP and CP groups are shown in Table 2. There was no difference in the sex ratio or pT classification among the groups, but there were significant differences in the pN and pStage classifications. The pN positive-rates (NP: 27.6%, SP: 89.5%, CP: 66.7%) varied, with a significantly lower rate in the NP group (p = 0.001). The pStage3/4 classification rate also varied (NP: 69.0%, SP: 94.7%, CP: 90.5%), with a significantly lower rate in the NP group (p = 0.010). The rate of recurrence or metastasis within 5 years after surgery was lowest in the CP group (NP: 27.6%, SP: 36.8%, CP: 14.3%), but with no significant difference among the groups (p = 0.183). The 5-year mortality rate after surgery was lowest in the CP group (NP: 34.5%, SP: 34.2%, CP: 9.5%), but the difference among the three groups was not significant (p = 0.089). However, in Kaplan-Meier analyses, the OS rate was found to be significantly higher in the CP group (CP vs. NP, p = 0.028; CP vs. SP, p = 0.042) (Fig. 1). There was similar tendency of the difference in the DF rates, although p values did not reach the significant level in Kaplan-Meier analysis (CP vs. NP, p = 0.149; CP vs. SP, p = 0.071) (Fig. 2).

**Table 2:** Characteristics and prognoses in patients with different postsurgical treatment

Item	NP group	SP group	CP group	P value^a^
	(n = 29)	(n = 38)	(n = 21)	
Sex, n(%)				
Female	11 (37.9)	11 (28.9)	6 (28.6)	0.689
Male	18 (62.1)	27 (71.1)	15 (71.4)	
pT, n(%)				
T1 + T2	10 (34.5)	7 (18.4)	5 (23.8)	0.319
T3 + T4a	19 (65.5)	31 (81.6)	16 (76.2)	
pN, n(%)				
N0 (includes cases with local resection only)	21 (72.4)	4 (10.5)	7 (33.3)	0.001
N+	8 (27.6)	34 (89.5)	14 (66.7)	
pStage, n(%)				
Stage1 + 2	9 (31.0)	2 (5.3)	2 (9.5)	0.010
Stage3 + 4a + 4b	20 (69.0)	36 (94.7)	19 (90.5)	
Recurrence or metastasis				
No	21 (72.4)	24 (63.2)	18 (85.7)	0.183
Yes	8 (27.6)	14 (36.8)	3 (14.3)	
5-year postsurgical mortality				
No	19 (65.5)	25 (65.8)	19 (90.5)	0.089
Yes	10 (34.5)	13 (34.2)	2 (9.5)	

^a^ Chi-squared test

### Mortality of OSCC patients with major risk by different postsurgical treatment methods

Mortality did not differ significantly with use of CDDP in univariate analysis (HR: 1.368, 95% CI: 0.603–3.103, p = 0.453) or multivariate analysis (HR: 1.112, 95% CI: 0.437–2.826, p = 0.824) (Table 3). Similarly, there was no difference in mortality based on radiotherapy in univariate analysis (HR: 0.797, 95% CI: 0.357–1.777, p = 0.579) or multivariate analysis (HR: 0.726, 95% CI: 0.297–1.766, p = 0.483) (Table 3). In contrast, mortality was significantly decreased by cetuximab treatment in univariate analysis (HR: 0.228, 95% CI: 0.054–0.968, p = 0.045) and was decreased with close to significance in multivariate analysis (HR: 0.233, 95% CI: 0.053–1.028, p = 0.054) (Table 3).

**Table 3:** Mortality of OSCC patients with major risk by different postsurgical treatment methods in a 5-year follow-up period (n = 88)

Factor	Univariate analysis					Multivariate analysis					
	Crude HR	95% CI			P value^a^	Adjusted HR	95% CI			P value^a^	
Cisplatin	1.368	0.603	-	3.103	0.453	1.112	0.437	-	2.826	0.824	
Radiation	0.797	0.357	-	1.777	0.579	0.726	0.297	-	1.776	0.483	
Cetuximab	0.228	0.054	-	0.968	**0.045**	0.233	0.053	-	1.028	0.054	

HR: Hazard ratio, CI: Confidence interval

^a^ Cox-proportional hazard model

## Discussion

We have used cetuximab to treat OSCC patients with major risk after clinical complete resection at our hospital since 2014. In this study, we conducted a retrospective cohort study to examine the effectiveness of cetuximab as a preemptive postsurgical therapy for OSCC patients with major risk, in other words, OSCC patients with invisible metastasis. The 5-year OS rate in patients who received the postsurgical treatment including cetuximab (CP group) was significantly higher than that in the other two groups (NP and SP groups). The 5-year DF rate was also higher in patients who received the postsurgical treatment including cetuximab (CP group), although p value did not reach significant level. We performed multivariate analysis to evaluate the effects of the main components of the treatment, because patients who received the postsurgical treatment including with cetuximab and standard postsurgical treatment sometimes received concomitant radiotherapy. Among CDDP, radiotherapy, and cetuximab, only cetuximab significantly contributed to improved survival by univariate analysis (crude HR:0.228, 95%CI:0.05–0.968, p = 0.045). cetuximab also showed the same tendency in multivariate analysis, although p value did not reach significant level (Adjusted HR: 0.233, 95%CI: 0.053–1.028, p = 0.054). Concurrent radiation with the administration of cetuximab might act as a confounding factor.

In the three groups in the study, the pN-positive and pStage3 + 4 rates differed significantly, with the lowest rates in patients who did not receive postsurgical treatment (NP group) (pN-positive rate: 27.6%, pStage3 + 4: 69.0%). This suggests that these patients may not have been indicated for postsurgical treatments, which suggests an influence of the clinicopathological characteristics on the judgment of surgeons with regard to the treatment. However, in patients who received postsurgical standard protocol (SP group), the pN-positive rate was significantly lower in patients who received postsurgical treatment including cetuximab (CP group) (SP: 89.0% vs. CP: 66.7%, p = 0.042 by Fisher exact test), but the pStage3 + 4 rate did not differ between the groups (SP: 94.7% vs. CP: 90.5%, p = 0.611 by Fisher exact test). These findings show that the clinicopathological evaluation of patients in CP group was not severe than those in the other two groups (NP and SP). In order to avoid the bias for selecting the patients in each group, a randomized controlled study should be conducted.

A large-scale clinical study in patients with head-and-neck SCC with recurrence or distant metastasis showed an additional effect of cetuximab over a combination of fluorouracil and a platinum agent (EXTREME study) [13]. An improved effect of BRT with cetuximab compared with RT alone in primary treatment for locally advanced head-and-neck SCC has also been reported [14]. However, studies that have established the consensus on the usefulness of cetuximab have mainly included patients who were difficult to treat with surgery. This treatment has also been investigated only as an alternative for patients with platinum-resistant tumors [15–17]. The NCCN guidelines recommend platinum-based chemotherapy, radiotherapy, or both as first-line treatment for patients with oral cancer with AFs after surgery. However, Palmer et al. found that radiotherapy + cetuximab was well tolerated and resulted in better long-term survival and less distant metastasis in a comparison of radiotherapy alone with radiotherapy + cetuximab for cutaneous squamous cell cancer of the head and neck in patients with high postsurgical risk factors [18]. These findings are similar to the results of our study.

The marked improvement in postsurgical survival with cetuximab in this study may be due to inhibition of the development of visible recurrence or metastasis from dormant cancer cells after surgery. The expression of EGFR in OSCC cells is elevated compared with that in colorectal or bladder cancer [19]. However, as shown in A431 cells, oral cancer cells express both full-length EGFR and EGFR lacking the intracellular domain [20, 21]. Therefore, EGFR is considered to be a signal transducer, as well as a tumor marker, in OSCC cells. In addition, proliferation of cancer cells including oral cancer is not always enhance by exogenous EGFR ligands, such as EGF and TGF-α, and such exogenous ligands sometimes inhibit growth of the cancer cells [22, 23]. Therefore, we do not think that antitumor effects of cetuximab are obtained by blocking EGFR signaling alone in patients with OSCC. Panitumumab, a complete human IgG2 antibody, is effective for colorectal cancer, but not for head and neck cancers, including oral cancer [24]. We have recently suggested that the main action of cetuximab in oral cancer may be immunological reaction, such as ADCC, rather than signaling blockade [11].

There are several limitations in the study, including the small sample size for definitive statistical analysis and the retrospective single-center design. Furthermore, as we mention above, there is some bias for selecting the patients in each group. Therefore, we are now planning to conduct a prospective randomized controlled study in a larger number of patients with consistent backgrounds at multiple facilities to validate our findings.

## Conclusions

The postsurgical treatment with cetuximab as a preemptive postsurgical therapy after complete surgical resection of a visible tumor is considerably effective for OSCC patients with major risk, in other words, invisible dormant metastasis.

## Data Availability

The datasets generated and/or analyzed during the current study are available from the corresponding author upon reasonable request.
